# The Psychological Mechanisms Underlying Solomon’s Paradox: Impact of Mood and Self-Transcendence

**DOI:** 10.3389/fpsyg.2022.901012

**Published:** 2022-07-22

**Authors:** Wentao Xu, Kaili Zhang, Fengyan Wang

**Affiliations:** ^1^Institute of Moral Education, Nanjing Normal University, Nanjing, China; ^2^School of Psychology, Nanjing Normal University, Nanjing, China

**Keywords:** Solomon’s paradox, wise reasoning, self-transcendence, PANAS, mood

## Abstract

Solomon’s paradox of wise reasoning, in which performance of wisdom differs when reasoning on an issue in one’s own life vs. another’s life, has been supported by robust evidence. However, the underlying psychological mechanism remains unclear. This asymmetry of wise reasoning may be explained by the different mindsets of self-transcendence when people reason about various conflicts (personal vs. others’), and mood should play a fundamental role. To explore this issue, three hundred ninety-nine participants were recruited to test a hypothesized model. The results supported the effect of Solomon’s paradox—that is, participants endorsed wise-reasoning strategies more strongly when resolving others’ social conflicts than their own. Further mediation analysis showed that the sequential mediation model was supported. Solomon’s paradox can be explained by the difference in positive affect and self-transcendence when reasoning about the two conflicts. This study directly verifies the mediating role of self-transcendence in Solomon’s paradox. At the same time, reasoning about personal affairs reduces individuals’ self-transcendence mindset, and positive affect can explain the differences. These results are helpful for understanding and effectively avoiding Solomon’s wisdom dilemma.

## Introduction

After ascending to the throne at the age of 20, King Solomon asked God for immense wisdom in a dream. The power of Solomon’s wisdom is evident in many famous stories; however, that wisdom did not help him cope well with problems in his personal. In fact, his profligacy and extravagance in his later years eventually led to the downfall of his dynasty.

This asymmetry in the performance of wisdom on issues in one’s own life and those in others’ is known as Solomon’s paradox (Grossmann and Kross, 2014). This is in line with Mickler and Staudinger’s (2008) classification of personal wisdom as one’s ability to consider one’s own life and general wisdom as insight into the lives of others from an observer’s perspective. No single person will necessarily have both types of wisdom. People may show wisdom regarding others’ life problems while being stuck in their problems; Solomon’s sound general wisdom helped him deal with others’ life problems effectively, but he lacked the personal wisdom to live his own life well (Staudinger and Glück, 2011; Staudinger, 2013). The stories of Solomon and the perspectives offered by prior research suggest that Solomon’s paradox may represent a fundamental and widespread social cognitive bias.

Expanding on this idea, empirical research has found in recent years that people are less likely to adopt and endorse multiple wisdom-related strategies when reasoning about personal issues than when thinking about others’ conflicts when numerous interpersonal conflicts, such as partner infidelity, are present. This phenomenon is present among both young and older adults, providing robust evidence for Solomon’s paradox (Grossmann and Kross, 2014; Huynh et al., 2017). Here, wisdom (reasoning) is defined as multiple wisdom-related reasoning strategies that help people cope with significant life challenges such as intellectual humility, dialectical thinking, compromise seeking, and perspective-taking (Baltes and Smith, 2008; Grossmann and Kross, 2014; Brienza et al., 2018).

Both young and old participants scored relatively low on all dimensions of wise reasoning when thinking about their personal conflicts, were less likely to adopt the views of the other person and third parties, were less aware of the limitations of their thoughts, and were less likely to seek compromise, among other things (Grossmann and Kross, 2014). Moreover, when they were personally involved in the conflict, participants were even less likely to recognize the effectiveness of these wise reasoning strategies for conflict resolution (Huynh et al., 2017). Solomon’s paradox exists in both the perspective and behavior dimensions of people.

### The Psychological Mechanism of Solomon’s Paradox

Why did King Solomon’s wisdom fail to guide him in controlling his own life? One reason is that people tend to adopt the first-person perspective when faced with their personal problems and the third-person perspective when thinking about others’ issues (Grossmann and Kross, 2014). Differences in cognitive processing under different perspectives lead to asymmetries in the performance of wise reasoning about one’s own and others’ life problems, especially in interpersonal conflict dilemmas involving self-threatening situations (Grossmann, 2017). Previous research has shown that, for the same life issues (career prospects for the unemployed during an economic recession) and general political matters (anticipated societal changes associated with one’s chosen candidate losing the 2008 United States presidential election), participants who reasoned from an ego-decentering perspective performed better in terms of intellectual humility and dialectical thinking relative to self-immersionists in terms of intellectual reasoning (Kross and Grossmann, 2012). Researchers further manipulated the reasoning perspective and found that self-distancing eliminated differences in people’s wise reasoning performance between their own and others’ conflicts (Grossmann and Kross, 2014).

In addition to perspective, a preregistration study found that pursuit of virtue moderates Solomon’s paradox. Individuals high in pursuit of virtue showed no differences in the endorsement of wisdom reasoning strategies between issues in one’s own and others’ lives (Huynh et al., 2017). Unlike previous hypothetical contexts (Baltes and Staudinger, 2000; Grossmann and Kross, 2014), Huynh et al. used a different conflict-initiation paradigm for event reconstruction. Participants were asked to recall an interpersonal conflict that occurred to them or a friend and rate the extent to which wise reasoning strategies could help them resolve the dispute (Huynh et al., 2017). The pursuit of virtue moderates Solomon’s paradox whereby high virtue-seekers endorses the effectiveness of wise reasoning strategies for conflict resolution to the same extent in their personal conflicts and others’ conflicts (Huynh et al., 2017). In both studies, the pursuit of virtue moderated the dimension of intellectual humility, which they suggested may be due to the self-transcendence embedded in virtue that enables people to see through the “illusions of one’s truth” (Huynh et al., 2017). Previous research has also supported the positive impact of self-transcendence as a developmental trait on wisdom personality (Le, 2011). However, no study has directly examined the role of self-transcendence in Solomon’s paradox.

### Self-Transcendence as a Mediator

Psychology’s focus on self-transcendence began with Viktor Frankl’s *Man’s Search for Meaning*. Frankl argued that, if people could devote themselves to a cause or love someone, such self-forgetfulness could lead to self-actualization (Frankl, 1946). Late in his career, Maslow also envisioned a stage of self-transcendence driven by transcendent values above self-actualization (Maslow, 1969; Koltko-Rivera, 2006). Self-transcendence is now empirically defined as the expansion or dissolution of ego boundaries and an increase in feelings of connectedness with a larger context (Reed, 1991; Levenson et al., 2005; Sortheix and Schwartz, 2017; Yaden et al., 2017; Fishbein et al., 2020).

Three perspectives currently exist on the relationship between wisdom and self-transcendence: self-transcendence as a contributing factor in the performance of wisdom (Le, 2011), as an aspect or subtype of wisdom (Achenbaum and Orwoll, 1991; Wink and Helson, 1997), or as central to the psychological process of wisdom (Aldwin et al., 2019, 2020). However, when considering wisdom as a situational attribute, there is apparent heterogeneity between self-transcendence and “excellence in social cognition” (wise reasoning, Grossmann et al., 2020) at both the trait and state levels.

In Solomon’s paradox, we argue that thinking about one’s personal social conflicts can directly inhibit one’s self-transcendent mindset, leading to poor wise reasoning performance. It has been argued that facing self-threat from interpersonal conflict causes people to naturally focus on their cognition and emotions (Jonas et al., 2014; Grossmann, 2017). Thinking about conflict inevitably reduces individuals’ situational awareness in the self-transcendence mindset. In particular, in the event reconstruction technique of wise reasoning measures, multiple means are used to ensure that individuals revert to the recalled conflict situation and that their cognitive and emotional involvement in the conflict is fully evoked (Brienza et al., 2018). Thus, self-threat from thinking about one’s personal conflict may reduce individuals’ situational self-transcendence whereas thinking about the conflicts of others does not or may even promote individuals’ self-transcendence mindset, which may be an important reason for Solomon’s paradox arising. For this reason, we propose our first hypothesis (H1), that self-transcendence is significantly lower when thinking about one’s own conflict problems than others’, and our second hypothesis (H2), that self-transcendence plays a mediating role in conflict type and wise reasoning.

### Mood as a Mediator

We also sought to examine the role of mood as differences in affect when thinking about different conflict problems may affect people’s endorsements of wise reasoning. Grossmann and Kross (2014) found that thinking about conflicts directly reduced positive affect, reinforced negative affect, and enhanced emotional arousal. In Huynh et al.’s (2017) study, positive affect positively predicted agreement with wise reasoning in both personal and others’ conflicts whereas the effect of negative affect was not significant. Also, positive affect was positively associated with wiser reasoning across studys in Grossmann et al. (2019). However, no study has examined differences in mood when thinking about the two conflicts and their roles in Solomon’s paradox. Numerous studies have shown that positive affect promotes creativity, openness, and exploration (Isen, 2000), which are associated with experiential wisdom exposure. In contrast, negative affect is associated with fine-grained processing and localized attention (Huntsinger et al., 2014). Accordingly, the self-relevance of conflicts may lead to different positive and negative affects following conflict reasoning, and that this difference may be a fundamental reason for Solomon’s paradox to arise (Grossmann and Kross, 2014).

At the same time, positive affect is positively associated with self-transcendence (Van Cappellen and Rimé, 2014; Garland and Fredrickson, 2019). According to the “broaden-and-build” theory, positive affect can expand attentional breadth and promote holistic perception, which can blur the boundaries between social groups through a state of “social broadening” and enhance people’s ability to transcend self-imposed limitations. “Social broadening” in social interactions can improve people’s sense of oneness beyond self-boundaries and increasing cooperative behavior between individuals and groups (Johnson and Fredrickson, 2005; Wiltermuth and Heath, 2009). Positive affect may play a fundamental role in the self-transcendent mindset when thinking about the two conflict issues; that is, positive affect also explains the effect of thinking about the type of conflict on self-transcendence. In summary, reactive emotional states may be the underlying psychological factor for the differences in self-transcendence and wise reasoning across conflicts. Low self-transcendence leading to poor wise reasoning performance may stem from experiencing less positive affect or more negative affect when thinking about one’s problems. Therefore, the hypothesized model also includes mood to form a sequential mediator model. Accordingly, we propose our third hypothesis (H3): Mood and self-transcendence play a sequential mediating role in the relationship between conflict type and wise reasoning.

### The Present Study

**FIGURE 1:**
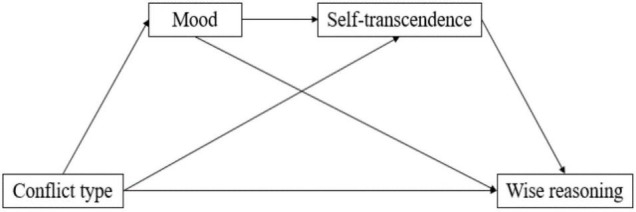
Hypothesized model.

This study sought to directly examine the mediating roles of mood and self-transcendence in Solomon’s paradox. We hypothesized that different conflicts could lead directly to differences in wise reasoning but would also impact wise reasoning through mood, self-transcendence, and the sequential mediation of the two. The overall hypothetical model is as shown in Figure 1.

## Materials and Methods

### Participants

For the sample size, the G*Power program recommended a sample of 120 participants per condition to achieve a statistical power of 0.80 (Huynh et al., 2017). But they only found a marginal effect of conflict type on the endorsement of wise reasoning and only intellectual humility was consistent across both studies with 356 participants. So, more participants were recruited online *via* MTurk to observe whether Solomon’s paradox can be observed in more subcomponents of wise reasoning. Each participant was compensated $0.50 (USD) for participating. To control the quality of participants’ responses, we excluded participants who failed attention-check questions such as “Sometimes I just click randomly to pass the survey as soon as possible.” Those who indicated that they were inattentive “most of the time” or more were excluded from the analysis. The final sample consisted of 399 participants (181 female, 218 male; mean age = 38.22 years, SD = 11.53).

### Procedure

Participants were recruited online to participate in this study under the “Daily Life Survey” theme. After collecting information on demographic variables, the participants were randomly assigned to a self-conflict group (*n* = 208) or an other’s conflict group (*n* = 191). The guide words used for event construction were identical to those used by Huynh et al. (2017):

“Think about a (vs. friends’) close relationship (family member, friend, or romantic partner) that is currently not going very well. For example, you (vs. your friend) may be fighting a lot lately or may not be talking as much as you (vs. they) used to. You are (vs. Your friend is) uncertain whether you (vs. he/she) will be able to continue to be as close to this person in the future.”

After event reconstruction, the participants were asked to report the type of relationship in which they or their friends had a conflict (e.g., romantic, familial, or friendship). In the self-conflict group, 28.8% of the conflicts were with romantic partners, 26.0% with family members, 41.3% with friends, and 3.8% in other relationships. In the others conflict group, 34.0% of the conflicts were with romantic partners, 14.7% with a family member, 49.2% with a friend, and 2.1% were other relationship conflicts. Participants were then asked to describe what led to their conflicts. To further ensure that participants reentered their conflicts, they were asked to imagine that the conflicts they recalled continued to go poorly and then describe their thoughts and feelings about this situation. This completed the manipulation of the type of conflict to reason, followed by measures of wise reasoning, positive and negative affect, self-transcendence, and emotional intelligence.

### Measures

#### Wise Reasoning

The 21-item Situated Wise Reasoning Scale (SWIS, Brienza et al., 2018) was used to assess participants’ wise reasoning endorsements. The scale contains five dimensions: (1) others’ perspectives, e.g., “Putting myself in the other person’s shoes”; (2) consideration of change and multiple ways situation may unfold, e.g., “Looking for different solutions as the situation evolved”; (3) intellectual humility/recognition of limits of knowledge, e.g., “Considered whether the other person’s opinions might be correct”; (4) search for a compromise/conflict resolution, e.g., “Tried my best to find a way to accommodate both of us”; and (5) view of the event through the vantage point of an outsider, e.g., “Tried to see the conflict from the point of view of an uninvolved person.” Participants reported on this scale from 1 (very useless) to 5 (very useful) how valuable each reasoning strategy would be if they were trying to resolve the conflict they had described earlier in the event-reconstruction session, with higher ratings indicating greater endorsement of the wise reasoning strategies. In addition to average the ratings for the 21 items as an overall measure of endorsement of wise reasoning (*M* = 3.61, *SD* = 0.65, α = 0.92), we also computed an average score for each individual subcomponent of wise reasoning to explore the effects of conflict type, positive and negative affect, and self-transcendence on the endorsement of each subcomponent.

#### Mood

The 20-item Positive and Negative Affect Schedule (PANAS, Watson et al., 1988) was used to assess participants’ state affect. They were asked to report their state affect when thinking about the corresponding conflicts on the scale from 1 (strongly disagree) to 5 (strongly agree), including 10 positive-affect items (e.g., “interested,” “excited”; *M* = 3.07, *SD* = 0.91, α = 0.87) and 10 negative-affect items (e.g., “disappointed,” “angry”; *M* = 2.49, *SD* = 0.87, α = 0.88). Average scores were computed separately for positive and negative affect.

#### Self-Transcendence

A modified version of Adult Self-Transcendence Inventory (ASTI, Levenson et al., 2005) was used to assess participants’ state, as opposed to trait, mindset of self-transcendence (“Considering the conflict you just recalled, to what extent do you agree with each item below?”). Participants rated their agreement with 10 items, using a scale from 1 (strongly disagree) to 4 (strongly agree), e.g., “My sense of self does not depend on other people and things.” The average score of all items was computed as a measure of self-transcendence (*M* = 2.98, *SD* = 0.38, α = 0.72).

#### Emotional Intelligence

The 16-item Emotional Intelligence Scale (WLEIS, Wong and Law, 2002) was used to assess participants’ emotional intelligence. The scale includes four factors: (1) self-emotion appraisal, e.g., “I really understand what I feel”; (2) others’ emotion appraisal, e.g., “I am a good observer of others’ emotions”; (3) use of emotion, e.g., “I am a self-motivated person”; (4) regulation of emotion, e.g., “I have good control of my own emotions.” The response format was a 7-point Likert-type scale ranging from strongly disagree to strongly agree. The average score of all items was computed as a measure of emotional intelligence (*M* = 5.35, *SD* = 0.83, α = 0.90).

## Results

### Preliminary Analyses

Gender differences in wise reasoning, self-transcendence, positive versus negative affect, and emotional intelligence were not significant. There was no interaction between gender and conflict type for any variable. Age was correlated with some variables to varying degrees (Table 1).

**TABLE 1:** Correlation matrix of age and other variables (*N* = 399).

Variables	1	2	3	4	5	6
1. Age	1					
2. Positive affect	–0.03	1				
3. Negative affect	−0.16**	0.22**	1			
4. Emotional intelligence	0.09	0.20**	−0.12*	1		
5. Self-transcendence	–0.01	0.25**	–0.02	0.50**	1	
6. Wise reasoning	−0.14**	0.34**	0.20**	0.27**	0.29**	1

*p < 0.05, **p < 0.01.

### The Main Effect of Conflict Type

Consistent with the hypotheses, participants found it more useful (Table 2) to use wise reasoning strategies to resolve friends’ conflicts (*M* = 3.73, *SD* = 0.58) than their own (*M* = 3.50, *SD* = 0.69), *F*(1, 398) = 12.21, *p* = 0.001, η_p_^2^ = 0.03, 90% CI = [0.12, 0.33], and the differences were significant for all five subcomponents (*p*s < 0.05). For the two most typical types (romantic and friendship), participants endorsed wise reasoning strategies as more useful for resolving a friend’s conflict than for resolving their own, *F*(1, 301) = 11.88, *p* = 0.001, η_p_^2^ = 0.04; and endorsed wise reasoning strategies as more useful for resolving romantic conflicts than friendship conflicts, *F*(1, 301) = 4.42, *p* = 0.036, η_p_^2^ = 0.01; but the interaction was not significant, *p* = 0.27. And when others’ perspectives and view of the event through the vantage point of an outsider were excluded from SWIS, participants still endorsed wise reasoning strategies as more useful for resolving a friend’s conflict than for resolving their own, *F*(1, 397) = 12.21, *p* = 0.001, η_p_^2^ = 0.03.

**TABLE 2:** The main impact of conflict type on wise reasoning and related variables.

Dependent variables	Personal conflicts (*M* ± *SD*)	Others’ conflicts (*M* ± *SD*)	*F*	η_p_^2^
Negative affect	2.47 ± 0.79	2.51 ± 0.95	0.16	–
Positive affect	2.99 ± 0.89	3.17 ± 0.91	4.06*	0.01
Interested	3.31 ± 1.35	3.60 ± 1.20	5.29*	0.01
Excited	2.50 ± 1.45	2.81 ± 1.44	4.52*	0.01
Enthusiastic	2.50 ± 1.32	2.83 ± 1.38	6.04*	0.02
Emotional intelligence	5.30 ± 0.88	5.40 ± 0.77	1.43	–
Self-transcendence	2.94 ± 0.38	3.03 ± 0.41	4.58*	0.01
Wise reasoning	3.50 ± 0.69	3.73 ± 0.58	12.21**	0.03
Others’ perspectives	3.42 ± 0.87	3.70 ± 0.80	11.23**	0.03
Consideration of change	3.59 ± 0.84	3.84 ± 0.67	10.68**	0.03
Intellectual humility	3.49 ± 0.83	3.67 ± 0.64	5.39*	0.01
Search for a compromise	3.67 ± 0.78	3.85 ± 0.69	6.13*	0.02
View of an outsider	3.29 ± 0.89	3.54 ± 0.81	8.08**	0.02

*p < 0.05, **p < 0.01.

Self-transcendence was significantly lower for participants thinking about personal conflicts (*M* = 2.94, *SD* = 0.38) than for others (*M* = 3.03, SD = 0.41), *F*(1, 398) = 4.58, *p* = 0.033, η_p_^2^ = 0.01, 90% CI = [0.02, 0.15]. Reasoning about personal conflict also led to lower positive affect, *F*(1, 398) = 4.06, *p* = 0.045, η_p_^2^ = 0.01, 90% CI = [0.03, 0.33]; differences were significant for interested, excited, and enthusiastic, and non-significant for all other affect, including any specific negative emotions.

Furthermore, the difference in emotional intelligence was not significant (*p* = 0.23) between the two conditions, indicating that it was not influenced by the independent variable manipulation. Regression analyses indicated that emotional intelligence positively predicted positive affect (β = −0.20, *p* < 0.001), self-transcendence (β = −0.50, *p* < 0.001), and wise reasoning (β = −0.27, *p* < 0.001) and negatively predicted negative affect (β = −0.12, *p* < 0.05), but the interaction between emotional intelligence and conflict type was not significant for other variables (*p*s > 0.05).

### Testing for Mediation

**FIGURE 2:**
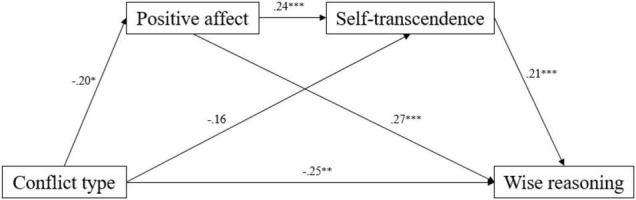
The sequential mediation of mood and self-transcendence. *p < 0.05, **p < 0.01.

In the hypothesis, we predicted that state affect and self-transcendence would mediate the relationship between conflict type and wise reasoning, so a mediation effect analysis was conducted (Figure 2 and Table 3). Results indicated a significant mediating effect of positive affect between conflict type and self-transcendence with an indirect effect size of −0.02, 95% CI = [−0.0479, −0.0020] and a non-significant direct effect [−0.1414, 0.0109], a significant mediating effect of positive affect between conflict type and wise reasoning with an indirect effect size of −0.04, 95% CI = [−0.0933, −0.0022] and a direct effect size of −0.18, 95% CI = [−0.3009, −0.0630], a significant mediating effect of self-transcendence between conflict type and wise reasoning with an indirect effect size of −0.04, 95% CI = [−0.1015, −0.0045] and a direct effect size of −0.18, 95% CI = [−0.3052, −0.0639], and a significant sequential mediating effect of positive affect and self-transcendence between conflict type and wise reasoning with an indirect effect size of −0.01, 95% CI = [−0.0240, −0.0008] and a direct effect size of −0.16, 95% CI = [−0.2758, −0.0427].

**TABLE 3:** Indirect pathways from conflict type to wise reasoning.

Indirect pathways	B	S.E.	95% CI
Conflict type → Positive affect → Wise reasoning	–0.04	0.02	–0.0933, –0.0022
Conflict type → Self-transcendence → Wise reasoning	–0.04	0.02	–0.1015, –0.0045
Conflict type → Positive affect → Self-transcendence → Wise reasoning	–0.01	0.01	–0.0240, –0.0008

For the two most typical types (romantic and friendship), the mediation effect analysis still indicated a significant sequential mediating effect of positive affect and self-transcendence between conflict type and wise reasoning with an indirect effect size of −0.01, 95% CI = [−0.0235, −0.0001]. And when others’ perspectives and view of the event through the vantage point of an outsider are excluded from SWIS, the mediation effect analysis still indicated a significant sequential mediating effect of positive affect and self-transcendence between conflict type and wise reasoning with an indirect effect size of −0.02, 95% CI = [−0.0736, −0.0023].

The mediating effects of positive affect and self-transcendence between conflict type and each component of wise reasoning were tested further. The results showed a significant sequential mediating effect on the other’s perspective with an indirect effect size of −0.01, 95% CI = [−0.0260, −0.0007] and a direct effect size of −0.22, 95% CI = [−0.38, −0.06], a significant sequential mediating effect on consideration of change and multiple ways the situation may unfold with an indirect effect size of −0.01, 95% CI = [−0.0275, −0.0009] and a direct effect size of −0.17, 95% CI = [−0.31, −0.03], a significant sequential mediating effect on intellectual humility/recognition of limits of knowledge with an indirect effect size of −0.01, 95% CI = [−0.0200, −0.0001] and a direct effect size of −0.12, 95% CI = [−0.26, −0.02], a significant sequential mediating effect on the search for a compromise/conflict resolution with an indirect effect size of −0.005, 95% CI = [−0.0230, −0.0005] and a non-significant direct effect, and a non-significant sequential mediating effect on view of the event from the vantage point of an outsider.

## Discussion

Although Solomon’s paradox of wise reasoning has received much attention (Kross and Grossmann, 2012; Grossmann and Kross, 2014; Huynh et al., 2017), the psychological mechanisms involved are still not quite precise. The present study directly examined the role of mood and self-transcendence and found that participants showed significantly lower self-transcendence when reasoning about personal conflicts than about those of others, supporting H1. Self-transcendence mediated the relationship between conflict type and wise reasoning, supporting H2. Positive affect and self-transcendence played significant sequential mediating role between conflict type and wise reasoning, partly supporting H3. The main contribution of this study is that these findings go deeper into the multiple occurrence mechanism of Solomon’s paradox. Additionally, the mediating role of positive affect provides theoretical guidance to avoid Solomon’s dilemma through emotion management.

### Solomon’s Paradox

Solomon’s paradox concerns the wisdom all people experience in life. Impaired wisdom performance in the face of personal life problems is a real problem that people should confront. Our results are consistent with previous findings that people not only use less wisdom-related cognitive strategies when coping with conflicts (Grossmann and Kross, 2014) but also do not recognize the effectiveness of wise reasoning strategies (Huynh et al., 2017). Unlike Huynh et al. (2017), who found significant differences only in some dimensions in terms of conflict types, our study found that Solomon’s paradox was represented on all subcomponents of wise reasoning, possibly because we adopted a between-subjects design (compared to Study 2) and obtained a larger sample size (compared to Study 1). Regarding the mechanisms involved, Grossmann (2017) explained this difference in terms of cognitive perspective when faced with different conflicts and provides indirect evidence with the moderating effect of self-decentering. In addition, Huynh et al. (2017) found that pursuit of virtue moderates Solomon’s paradox, suggesting that psychological factors may exist beyond perspective preference.

### The Mediating Role of Self-Transcendence

While the relationship between wisdom and self-transcendence is undeniable, the positioning of self-transcendence in different wisdom theories varies widely. For example, Aldwin et al. (2020) viewed self-transcendence as the core of wisdom or even wisdom itself while Grossmann et al.’s (2020) contextually oriented generic model of wisdom had difficulty accommodating self-transcendence in a rounded way. When wisdom is viewed as a personality trait, we argue that self-transcendence should be included in its complex construct. In contrast, if wisdom is considered a contextual manifestation of wisdom reasoning, both trait- and state-level self-transcendence should be subsumed as influences.

Similar to Le’s (2010) study in which trait self-transcendence positively predicted wisdom personality, the present study found that simply thinking about one’s interpersonal conflict reduced self-transcendent mindset, which led to poor performance in wisdom reasoning, and that self-transcendence mediated the relationship between conflict type and wisdom reasoning. This not only creatively develops a new paradigm of self-transcendence manipulation but also directly explains the occurrence mechanism of Solomon’s paradox and expands the depth and breadth of research in both fields, which should be integrated at theoretical and empirical levels in the relationship between the two in the future.

### The Mediating Role of Mood

Early theories of wisdom paid little attention to the importance of emotions with only Ardelt’s (2003) three-dimensional view of wisdom incorporating affect as a core dimension in the wisdom construct. In recent years, researchers have begun to explore the relationship between emotions and emotion-related psychological characteristics and wisdom, such as Thomas et al.’s (2019) San Diego Wisdom Inventory, which includes emotion regulation as one of six dimensions, Schneider et al.’s (2021) finding that emotional intelligence positively predicts both trait- and state-level wisdom, and the MORE life experience model, which considers emotion regulation and empathy to be important resources of wisdom (Glück et al., 2019; Glück et al., 2013). A longitudinal follow-up study by Grossmann et al. (2019) found a positive correlation between wise reasoning and emotional diversity rather than intensity.

Our study supports the positive predictive role of positive affect and emotional intelligence on self-transcendence and wise reasoning, which suggests an essential link between wisdom and emotions and related abilities (Grossmann et al., 2019; Schneider et al., 2021); on the other hand, the mediating role of positive affect in Solomon’s paradox was found, which suggests the complexity of the underlying mechanisms, where essential positive affect suppression beyond the cognitive perspective and self-transcendent mindset can lead directly to impaired wise reasoning endorsement. These results point to a theoretical path to improving wisdom through emotion management.

However, no significant differences between conditions were observed in any specific negative emotions. By comparison, Huynh et al.’s (2017) study also revealed quite low negative affect (*M* = 1.93, *SD* = 0.81, α = 0.91) and relatively higher positive affect (*M* = 3.26, *SD* = 0.80, α = 0.89). This may be an inherent defect of event reconstruction technology: After all, the conflicts recalled has passed.

### Limitation and Theoretical Implication

The main limitation of this study was that the effect sizes of the main findings were relatively small. The effect size for Solomon’s paradox was η_p_^2^ = 0.05–0.25 in Grossmann and Kross (2014) and η_p_^2^ = 0.01–0.05 in Huynh (2017), and the effect size was η_p_^2^ = 0.03 in our study. Overall, our results generally agree with those of Huynh (2017), but both are significantly smaller than the effect sizes derived by Grossmann and Kross (2014). One possible explanation is that Grossmann and Kross (2014) used three self-assessment questions and one objective scoring indicator to measure wise reasoning (Study 1: η_p_^2^ = 0.25). The effect sizes decreased sharply when the number of questions was increased to just seven (Study 2: η_p_^2^ = 0.12, Study 3: η_p_^2^ = 0.05) whereas our study and Huynh (2017) used a 19/21-question situational wise reasoning scale; robust measures of standardized scales may have more difficulty capturing Solomon’s paradox. Furthermore, Grossmann and Kross (2014) examined the use of wise reasoning strategies. In contrast, both our study and Huynh (2017) measured the endorsement of wise reasoning strategies, and the subtle differences between the two may also explain the difference in effect sizes. However, this also suggests that the mere difference in endorsing wise reasoning strategies of η_p_^2^ = 0.01–0.05 may translate into a η_p_^2^ = 0.05–0.25 difference in wise reasoning. These findings provide a deeper understanding of the cognitive and behavioral robustness of Solomon’s paradox.

Furthermore, major information difference between what we know about personal conflicts and those of friends may be a confounder in Solomon’s paradox when event-reconstruction is used. Fictitious conflicts used in Grossmann and Kross (2014) provide almost the same amount of but quite thin information for both conditions of personal and others’ conflicts. The event-reconstruction technology makes up for the lack of information, but raised a new problem of potential asymmetry of information in both conditions. To a large extent, this asymmetry may be an important reason for Solomon’s paradox in daily lives. However, future research should take measures to separate and investigate or control this confounding variable for a deeper understanding of Solomon’s paradox. Another limitation is that ethnic backgrounds and native languages are not included in this study, which may impair the measurements.

## Conclusion

This study examined the differences in mood, self-transcendence, and wise reasoning under different conflict types, directly tested the psychological mechanisms of Solomon’s paradox through a sequential mediation model, and verified the mediating role of positive affect and self-transcendence between conflict types and wise reasoning. These findings help deepen the academic understanding of the underlying mechanism of Solomon’s paradox but also provide a theoretical path based on emotion management to avoid Solomon’s dilemma.

## Data Availability Statement

The raw data supporting the conclusions of this article will be made available by the authors, without undue reservation.

## Ethics Statement

The studies involving human participants were reviewed and approved by Ethics Board of the Nanjing Normal University. The ethics committee waived the requirement of written informed consent for participation.

## Author Contributions

FW and WX designed the research. WX and KZ carried out the research and analyzed the data. WX wrote the manuscript. All authors contributed to the article and approved the submitted version.

## Conflict of Interest

The authors declare that the research was conducted in the absence of any commercial or financial relationships that could be construed as a potential conflict of interest.

## Publisher’s Note

All claims expressed in this article are solely those of the authors and do not necessarily represent those of their affiliated organizations, or those of the publisher, the editors and the reviewers. Any product that may be evaluated in this article, or claim that may be made by its manufacturer, is not guaranteed or endorsed by the publisher.
